# Correction: Microfluidic ELISA employing an enzyme substrate and product species with similar detection properties

**DOI:** 10.1039/d2an90045a

**Published:** 2022-06-08

**Authors:** Basant Giri, Yukari Liu, Fidelis N. Nchocho, Robert C. Corcoran, Sakur Mahmud, Debashis Dutta

**Affiliations:** Department of Chemistry, University of Wyoming Laramie WY 82071 USA ddutta@uwyo.edu

## Abstract

Correction for ‘Microfluidic ELISA employing an enzyme substrate and product species with similar detection properties’ by Basant Giri *et al.*, *Analyst*, 2018, **143**, 989–998, https://doi.org/10.1039/C7AN01671A.

The authors regret the omission of one of the authors, Sakur Mahmud, from the original manuscript. The corrected author list is as shown above.

The Royal Society of Chemistry apologises for these errors and any consequent inconvenience to authors and readers.

## Supplementary Material

